# In Situ and Operando Characterizations of 2D Materials in Electrochemical Energy Storage Devices

**DOI:** 10.1002/smsc.202000076

**Published:** 2021-03-10

**Authors:** Caixia Meng, Pratteek Das, Xiaoyu Shi, Qiang Fu, Klaus Müllen, Zhong-Shuai Wu

**Affiliations:** ^1^ State Key Laboratory of Catalysis Dalian Institute of Chemical Physics The Chinese Academy of Sciences Dalian 116023 China; ^2^ University of Chinese Academy of Sciences Beijing 100049 China; ^3^ Max-Planck-Institut für Polymerforschung Ackermannweg 10 Mainz 55128 Germany; ^4^ Dalian National Laboratory for Clean Energy Chinese Academy of Sciences 457 Zhongshan Road Dalian 116023 China

**Keywords:** 2D materials, in situ and operando, lithium batteries, supercapacitors

## Abstract

The urgent need of modern society for portable energy‐consuming devices has boosted the development of high‐power supercapacitors and high‐energy batteries. Major prerequisites for augmenting wider practical applications are advanced electrode materials and deeper insights into the underlying electrochemical processes. Owing to their unique physicochemical properties, 2D materials, such as graphene, transition metal carbides, nitrides, and dichalcogenides, hold special promise. Characterizing their behavior under real operating conditions (operando) or at the site of operation (in situ) without disassembling the device helps uncover essential kinetic information, which may, otherwise, be lost. By identifying both harmful and beneficial mechanisms, these in situ and operando characterization techniques allow researchers to alleviate critical issues in existing materials and develop new materials with enhanced properties. Herein, a brief introduction including the preparation and the electrochemical energy storage application of 2D materials is first presented. The main concern, thereby, is the influence of preparation methods on the resulting electrode structure and electrochemical performance. Then, the electrochemical mechanisms underlying the operation of supercapacitors and lithium batteries are discussed in detail. Finally, the perspective and future direction of applying the in situ and operando techniques to model 2D materials in revealing some important processes are highlighted.

## Graphical abstract

In‐depth understanding of electrochemical processes with the assistance of in situ and operando characterization is key for electrochemical energy storage. The latest advances of the most promising 2D materials applied in energy storage are reviewed, and the current understanding about their charge storage and degradation mechanisms inferred from relevant in situ and operando techniques is illustrated.

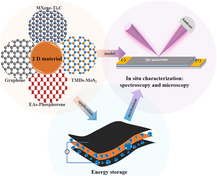

## 1 Introduction

The severe energy and environmental crisis faced by modern society has greatly increased the demand for renewable energies, such as wind, solar, and biomass. Energy transformation must go hand in hand with energy storage, in particular for intermittent renewable sources.^[^ ^1^ ^]^ Electrochemical energy storage devices offer enormous advantages due to high‐efficiency power grids and environmentally friendly operation.^[^ ^2^ ^]^ Among the energy storage devices, lithium batteries possess high energy density and high working voltage,^[^ ^3^ ^]^ whereas supercapacitors offer high power density and long cycle life, and both belong to the most promising candidates toward future energy technologies.^[^ ^4^ ^]^ Decreasing weight and size, increasing the cycle durability, maintaining safety, and minimizing cost have stood out as targets of world‐wide research and development.^[^ ^5^ ^]^ In the search for high‐performance electrodes,^[^ ^6^ ^]^ 2D materials, such as graphene, MXene, and transition metal dichalcogenides (TMDs), have attracted particular attention due to their various distinct merits.^[^ ^7^, ^8^, ^9^, ^10^ ^]^ In particular, the electron confinement in two dimensions of ultrathin 2D materials imparts them with unprecedented electronic characteristics, rendering them appealing candidates for energy storage applications.^[^ ^11^ ^]^ Given their high specific surface area, 2D materials enable full utilization of available sites of active electrode materials.^[^ ^12^ ^]^ Furthermore, the exposed contact area is significantly enhanced between the electrodes and the electrolytes, leading to short ion transport pathway.^[^ ^13^ ^]^ The interlayer spacings of many few‐layer and stacked 2D materials offer excellent ion transport channels for various ion‐intercalation energy storage systems. Besides, their structural flexibility, tunable and accessible surface area, and highly exposed active sites make them useful for flexible and smart devices.^[^ ^14^ ^]^

Another element of research toward improving device performance is the real‐time observation of electrochemical processes, because this approach offers insights into the underlying charge storage and degradation mechanisms, and could also allow one to monitor structural changes of the materials and their interfaces upon operation.^[^ ^15^, ^16^ ^]^ The technique of choice is so‐called in situ and operando characterization, where “in situ” means “at the site of the electrode at work” and “operando” implies the experiments under true operating conditions.^[^ ^17^ ^]^ Ex situ characterization is commonly used, but, to some extent, misses key information, because it is normally used after the electrochemical processes. Such an analysis can be further complicated, because the recovered materials are often sensitive to air and moisture. In contrast, in situ and operando monitoring presents obvious advantages such as the continuous observation of specific running process occurring at the selected areas of interest under true working conditions.

In this review, we focus on the latest advances in the application of 2D materials for electrochemical energy storage, seeking an in‐depth understanding of electrochemical processes with the assistance of in situ and operando characterization. Recent progress in the preparation and electrochemical energy storage applications of 2D materials are summarized. Meanwhile, the complicated reaction mechanisms in supercapacitors and lithium batteries are thoroughly discussed. The representative studies of the most frequently applied in situ and operando techniques to model 2D materials in revealing some important processes are particularly highlighted, and finally, the major current challenges and future opportunities are discussed.

## 2 2D Materials

**Figure 1:**
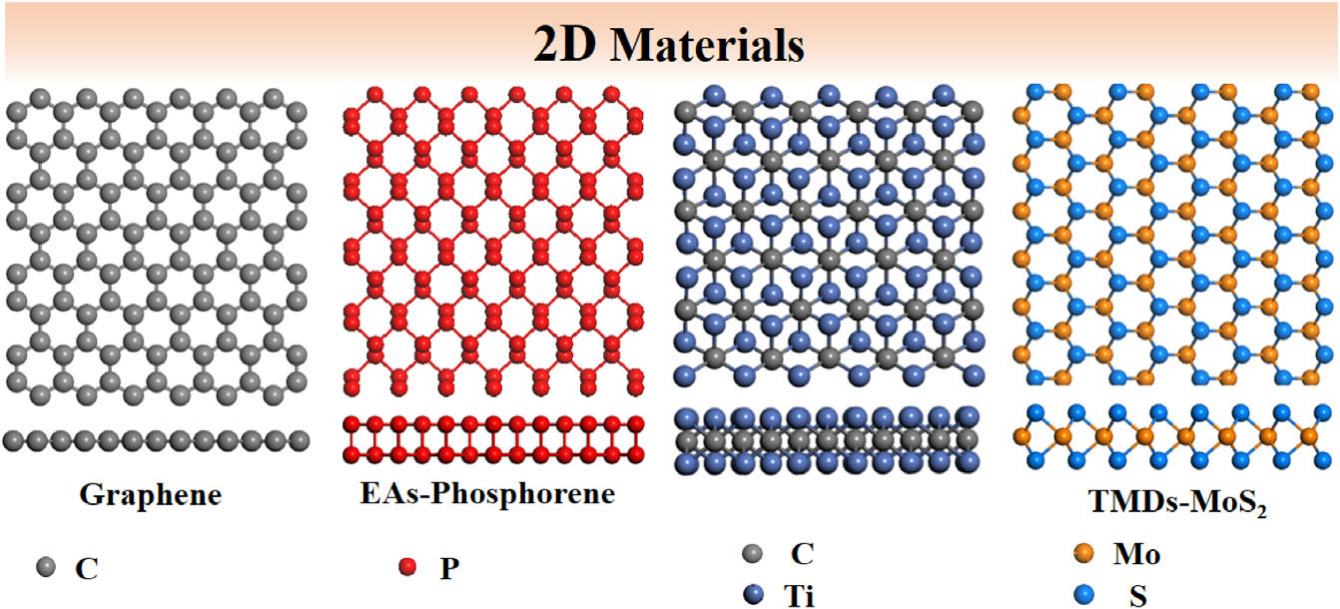
Typical structures of common 2D materials.

A variety of novel 2D materials with mono or several atom thick layers have been developed since the successful preparation of graphene by scotch‐tape micromechanical cleavage.^[^ ^18^ ^]^ As shown in **Figure** **1**, the blooming field of 2D materials comprises different structure types and compositions, such as elemental analogs of graphene (EAs), MXenes, TMDs, covalent organic frameworks (COFs), and metal organic frameworks (MOFs), among which EAs, MXenes, and TMDs are introduced preferentially here.^[^ ^19^, ^20^ ^]^ Similar to graphene, EAs contain one kind of element, mostly from Groups IV or V of the periodic table. The ones composed of Group IV elements, such as silicene, germanene, and stanene, have the same electronic configurations as graphene.^[^ ^20^ ^]^ In their stable states, the atoms arrange in a buckled hexagonal structure, unlike graphene, where all carbon atoms are located in a single plane with a perfect hexagonal honeycomb.^[^ ^21^ ^]^ The sheets are predicted to possess massless Dirac fermions, thereby displaying potential use in photoelectronic devices, similar to that of graphene.^[^ ^22^, ^23^ ^]^ Phosphorene, arsenene, antimonene, and bismuthene whose elements belong to Group V are mostly exfoliated from their original bulk counterparts with their electronic properties depending on the number of layers.^[^ ^24^ ^]^ MXenes are commonly described by the general formula M_*n*+1_X_*n*_T_*z*_, where M is an early transition metal atom, X is carbon and/or nitrogen, and T_*z*_ represents surface terminated functional groups (−OH, −O, or −F).^[^ ^25^ ^]^ The high intrinsic electronic/ionic conductivities of MXenes render them particularly promising electrode materials.^[^ ^26^, ^27^ ^]^ TMDs follow an MX_2_ structure, wherein M is a transition metal element (such as W, Mo, and Ti), and X is a class of chalcogen elements (such as S, Te, or Se).^[^ ^28^ ^]^ TMDs can adopt semiconducting, metallic, and even superconducting properties depending upon their chemical composition and layer structures.^[^ ^29^ ^]^ The 2D TMDs exhibit promising applications in electronics, optoelectronics, and energy technologies as a result of their excellent mechanical strength, optical transparency, and tunable electronic characteristics.^[^ ^30^ ^]^

### 2.1 Preparation of 2D Materials

**Figure 2:**
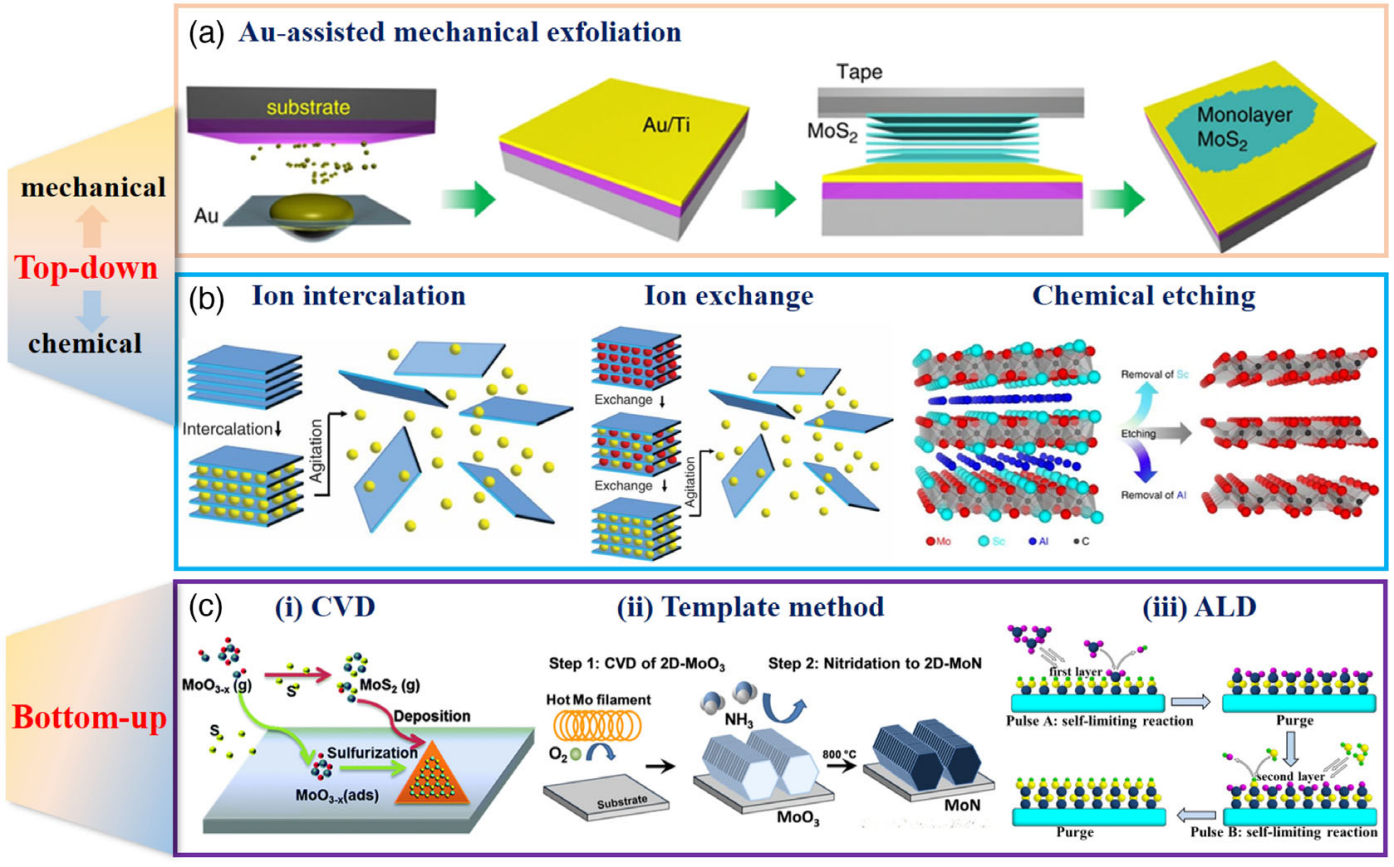
Typical fabrication strategies of 2D materials. a) Schematic description of Au‐assisted mechanical exfoliation. Reproduced under the terms of the CC‐BY 4.0 license.^[^ ^35^ ^]^ Copyright 2020, The Authors, published by Springer Nature. b) Schematic description of main chemical delamination methods. (Ion intercalation and Ion exchange) Reproduced with permission.^[^ ^38^ ^]^ Copyright 2013, American Association for the Advancement of Science. (Chemical etching) Reproduced under the terms of the CC‐BY 4.0 license.^[^ ^39^ ^]^ Copyright 2017, The Authors, published by Springer Nature. c) General introduction to the bottom‐up synthesis. i) CVD growth of MoS_2_ by the reaction of MoO_3‐*x*_ and S. Reproduced with permission.^[^ ^133^ ^]^ Copyright 2015, The Royal Society of Chemistry. ii) Schematic diagram of the two‐step process of synthesizing 2D δ‐MoN layers. Reproduced with permission.^[^ ^134^ ^]^ Copyright 2017, American Chemical Society. iii) Illustration of the ALD process. Reproduced with permission.^[^ ^135^ ^]^ Copyright 2015, American Chemical Society.

The controllable and reliable synthesis of high‐quality 2D materials is the key step for materials research and all applications derived thereof. At present, the emerging preparation methods can be categorized as “top‐down” and “bottom‐up”. The term top‐down refers to a process where a layered bulk material or powder is exfoliated into ultrathin 2D counterparts,^[^ ^31^ ^]^ which can be achieved by either mechanical or chemical delamination. While peeling with scotch tape was initially used as the primary technique to produce graphene, the procedure can also be achieved by ball milling,^[^ ^32^ ^]^ shearing,^[^ ^33^ ^]^ and ultrasonication.^[^ ^34^ ^]^ Recently, a universal gold‐assisted mechanical exfoliation method has been proposed, which yields high‐quality monolayer materials, as shown in **Figure** **2**a. Therefore, the adhesion between the 2D crystal is sufficient to overcome the interlayer attraction and facilitate exfoliation of monolayers from layered crystals.^[^ ^35^, ^36^, ^37^ ^]^ Examples of chemical delamination routes include ion intercalation, ion exchange, and chemical etching (Figure 2b). Naturally, ion intercalation increases the spacing between layers and weakens the interlayer interaction.^[^ ^38^ ^]^ Ion exchange, often with larger ions, occurs in some layered compounds containing intercalated ions.^[^ ^38^ ^]^ Wet chemical etching is the common method to obtain different types of MXene by selectively etching the multilayered precursor.^[^ ^39^ ^]^ Dependent of the type of etchant, the surfaces of the produced 2D layered materials are often decorated with various functional groups. The 2D materials generated via mechanical exfoliations possess high quality and purity, which are ideal for the fundamental research. However, the production yield is low, and the size, thickness, and shape of the as‐obtained 2D materials are difficult to control. Alternatively, high‐yield and massive production can be achieved through chemical delamination, whereas the thickness and the lateral sizes remain challenging. It should be noted that the top‐down methods are mainly applicable to yield those 2D materials derived from their layered bulk crystals.

The bottom‐up method is, in contrast, more suitable for the synthesis of large‐area 2D materials. One of the most commonly used bottom‐up protocols is the chemical vapor deposition (CVD) technique, in which volatile precursors react (or decompose) when they come in contact with a heated substrate. The substrate may contain preliminary templates that can be used to control the morphology or structure of the resultant film, in which case it is called the template‐assisted CVD. Another variant of the CVD method is atomic layer deposition (ALD), which, in essence, involves the reaction of one precursor with the substrate until an ultrathin layer is formed, followed by the second precursor's reaction with this layer in a self‐limiting manner. Figure 2c shows the processes involved in these techniques by taking the example of TMDs. Although these methods are promising to produce 2D materials with high crystal quality, tunable thickness, and scalable size, the requirements of high temperature and specific substrates hamper their practical applications. Through numerous efforts on the synthesis of a wide range of 2D materials, the universal mass production methods are still highly urgent to control the homogeneous morphology with atomic thickness, defined shape, and lateral size.^[^ ^40^ ^]^

### 2.2 2D Materials for Supercapacitors

**Figure 3:**
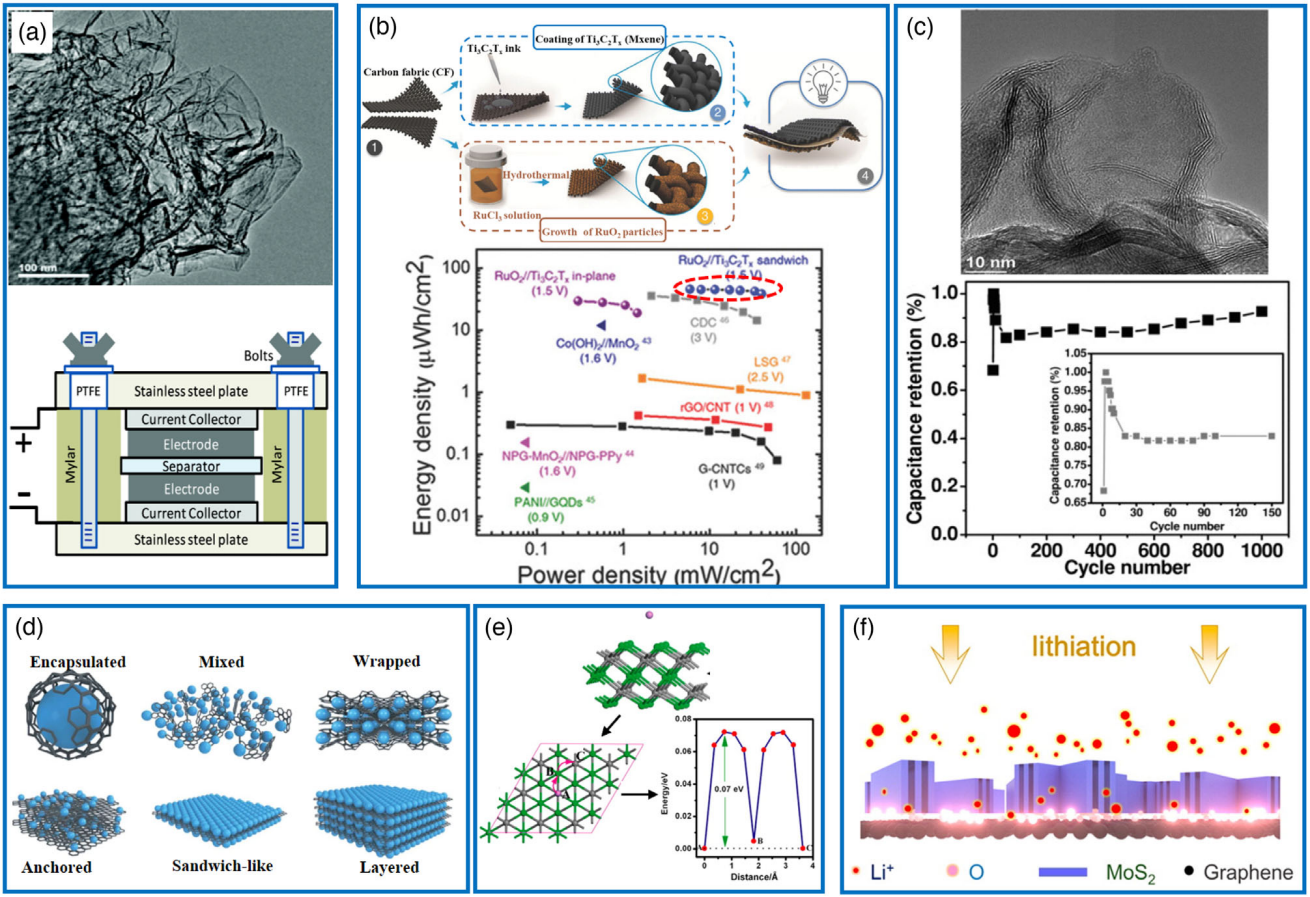
Typical 2D materials for supercapacitors and batteries. a) Top: TEM image of single graphene sheet. Bottom: Schematic description of a supercapacitor cell with graphene electrode. Reproduced with permission.^[^ ^50^ ^]^ Copyright 2008, American Chemical Society. b) Top: Schematic illustration of the fabrication of an asymmetric supercapacitor. Bottom: Ragone plot showing areal energy and power densities of a RuO_2_//Ti_3_C_2_T_*x*_ device compared with other advanced microsupercapacitors. Reproduced with permission.^[^ ^54^ ^]^ Copyright 2018, Wiley‐VCH. c) Top: SEM image of MoS_2_ nanosheets. Bottom: The cycle performance of the MoS_2_ film‐based microsupercapacitor. Reproduced with permission.^[^ ^55^ ^]^ Copyright 2013, Wiley‐VCH. d) Schematic description of the different graphene‐based composite electrode materials. Reproduced with permission.^[^ ^57^ ^]^ Copyright 2017, The Royal Society of Chemistry. e) Schematic representation of the considered migration paths and barriers for Li diffusion on Ti_3_C_2_. Reproduced with permission.^[^ ^60^ ^]^ Copyright 2012, American Chemical Society. f) Schematic representation illustrating lithium‐ion diffusion paths in the MoS_2_/G electrode. Reproduced with permission.^[^ ^64^ ^]^ Copyright 2016, American Chemical Society.

Although supercapacitors, in general, offer very high charge/discharge rates compared with batteries, a huge difference may be seen in the energy density and rate performance across different materials.^[^ ^41^ ^]^ This difference arises mainly from the mechanism in which the active material interacts with the electrolyte and from the architecture of the device itself. Conceptually, supercapacitors can be divided into electrical double‐layer capacitors (EDLCs), pseudocapacitors, and hybrid supercapacitors (e.g., lithium‐ion supercapacitors and battery supercapacitors) depending on the charge storage mechanism in either of the electrodes.^[^ ^42^, ^43^ ^]^ EDLCs possess electrodes that store electrical energy through rapid physical adsorption of ions.^[^ ^44^ ^]^ Pseudocapacitive electrodes involve reversible Faradaic reaction at the interface of the electrodes and electrolyte. Hybrid supercapacitors combine the physical adsorption on one electrode with Faradaic redox reactions on the other, thus achieving better rate capability than pseudocapacitors and better energy density than EDLCs. Carbon‐based materials are the most common EDLCs electrodes,^[^ ^45^, ^46^, ^47^ ^]^ and graphene or reduced graphene oxide materials have attracted huge attention due to their high specific surface.^[^ ^48^, ^49^ ^]^ As demonstrated in **Figure** **3**a, a supercapacitor based on chemically modified graphene can exhibit high performance with the specific capacitances up to 135 and 99 F g^−1^ in aqueous and organic electrolytes, respectively.^[^ ^50^ ^]^

While MXenes have been broadly applied in supercapacitors,^[^ ^8^, ^51^, ^52^ ^]^ the small voltage window of the MXene‐based symmetric supercapacitors in aqueous electrolytes, resulting from possible oxidation at high potentials, limits their energy density. To utilize their full value, combination with other materials has been applied.^[^ ^53^ ^]^ Jiang et al. designed pseudocapacitive supercapacitors by combining an MXene (Ti_3_C_2_T_*x*_) negative electrode with a RuO_2_ positive electrode, both of which were deposited on a conducting carbon fabric (top image in Figure 3b).^[^ ^54^ ^]^ This asymmetric device offered a broader potential window of 1.5 V, leading to an increased energy density of 37 μW h cm^−2^ at a power density of 40 mW cm^−2^. This performance is superior to most reported microsupercapacitors (Figure 3b).

The 2D TMDs such as MoS_2_ nanosheets have delivered capacitive performance comparable to those of carbon‐based materials (Figure 3c).^[^ ^55^ ^]^ The octahedral 1 T metallic phase of TMDs offers particularly promising supercapacitor applications as a result of their higher electrical conductivity compared with that of semiconducting 1H/2H phase. Monolayer 1 T MoS_2_ sheets, thus, have been fabricated and used as flexible electrode by Chhowalla and co‐workers.^[^ ^56^ ^]^ The packed supercapacitor from this flexible electrode can intercalate ions with extraordinary efficiency and yield volumetric capacitances ranging from 400 to 700 F cm^−3^ in various aqueous electrolytes with high stability (95% capacity retention after 5000 cycles in organic electrolytes).

### 2.3 2D Materials for Batteries

In general, the key structures of graphene/metal oxide composite electrode materials are categorized by six types of encapsulated, mixed, wrapped, anchored, sandwich‐like, and layered, as shown in Figure 3d. It is noted that the presence of graphene in these composite electrodes can greatly prevent the volume change of metal oxides during the charge/discharge process, accelerate the electron transport, and improve Li^+^ diffusion, resulting in high rate capability and long‐term cyclability.^[^ ^57^, ^58^, ^59^ ^]^ Quite recently, MXenes decorated with various functional groups have also been used in lithium batteries owing to their high capacity and easily tunable chemical and structural features. Ti_3_C_2_, one of the representative MXene materials, was identified as a promising anode material for Li ion batteries, because it not only possessed a high theoretical capacity of 320 mAh g^−1^, comparable to that of graphene, but also disclosed much lower barriers (0.07 eV) for Li diffusion than observed for anatase TiO_2_ (0.35–0.65 eV) (Figure 3e).^[^ ^60^ ^]^ However, the possible influence of surface groups (such as −O, −OH, and −F) on performance needs deeper consideration. So far, the oxygen functional groups on MXene surfaces have been reported to facilitate a higher capacity than their counterparts.^[^ ^51^, ^61^ ^]^

As expected from their unique layered structures, the 2D TMDs displayed remarkable performances in lithium batteries as a consequence of rapid ion insertion and extraction processes. Several studies have reported significantly reduced barriers of ion diffusion and improved adsorption energies by comparison with their bulk counterparts, leading to higher energy and power densities, as well as better cycling performance.^[^ ^62^, ^63^ ^]^ A delicate nanostructure with MoS_2_ layers grown vertically on graphene nanosheets (MoS_2_/G) has been reported as the anode material of lithium batteries (Figure 3f).^[^ ^64^ ^]^ Driven by the enhanced electron transport rate as a result of the interfacial interaction of the C—O—Mo bonds and by the buffering of volume changes afforded by graphene substrate, the rate performance and cycle life were improved significantly (1077 mAh g^−1^ at 100 mA g^−1^ after 150 cycles).

## 3 Fundamental Understanding of Electrochemical Processes

With the advances in preparation and application of 2D materials, more attention has been paid to the dynamic electrochemical processes underlying the operation of energy storage and conversion systems.^[^ ^65^, ^66^ ^]^ This section provides a brief introduction of some fundamental, but strongly debated mechanistic concepts decisive for the performance of supercapacitors and lithium batteries.

### 3.1 Fundamental Electrochemistry in Supercapacitors

**Figure 4:**
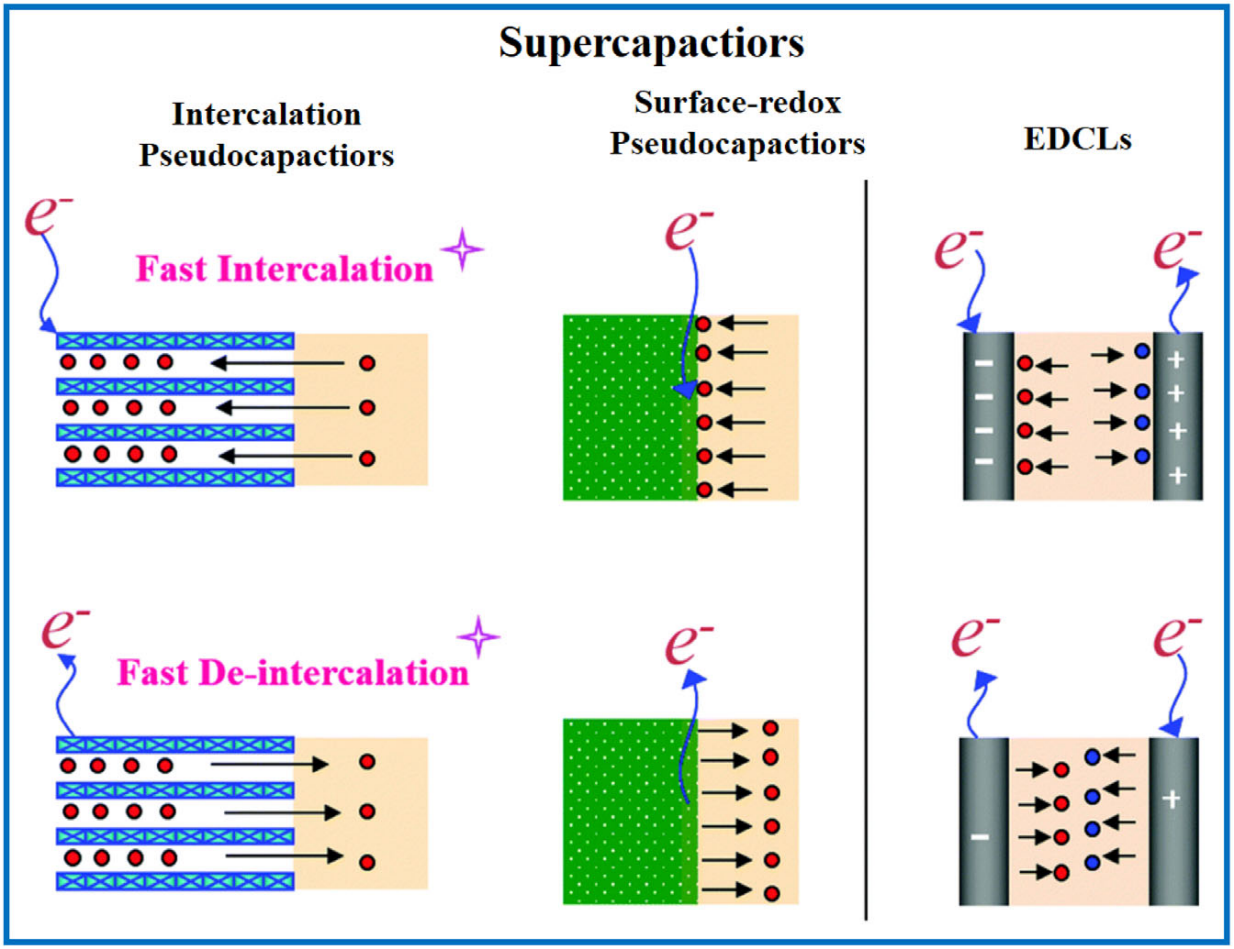
Schematic description of different charge storage mechanisms of supercapacitors. Charge storage mechanism of a supercapacitor. Reproduced with permission.^[^ ^68^ ^]^ Copyright 2016, The Royal Society of Chemistry.

As introduced earlier, EDLCs store charge via reversible adsorption–desorption of electrolyte ions onto electrodes (**Figure** **4**). Charge separation occurs upon polarization at the electrode–electrolyte interface, resulting in a Helmholtz layer depicted as double‐layer capacitance (*C*),^[^ ^67^ ^]^ $C=\frac{{\text{ε}}_{0}{\text{ε}}_{r}A}{d}$, where *ε*_0_ is the dielectric constant of vacuum, *ε*_r_ is the electrolyte dielectric constant, *A* is the surface area of the electrode, and *d* is the effective thickness of the double layer. Quite logically, when considering the role of *A*, attempts at enhancing the double‐layer capacitance would commonly use larger specific surface areas of electrodes.^[^ ^68^ ^]^ A higher specific surface area is supposed to arise from increased porosity. On the other hand, the pore size should be about twice the ion size to allow covering the pore walls. Thus, decreasing the pore size would lead to a high specific surface area. At the same time, however, fewer ions might be allowed to enter the pore. Experimental reports have proved this intuitive reasoning wrong, because the specific capacitance appeared to increase with decreasing pore size below a certain threshold.^[^ ^69^, ^70^ ^]^ It was speculated that the small pores caused a desolvation of the ions and reduced the distance between the charge centers of the electrolyte–electrode interface, thus causing an increase in capacitance. As for supercapacitors based on ionic‐liquid electrolytes, experimental and theoretical studies have indicated that the capacitance reached its maximum when the pore size matched the ion size.^[^ ^71^ ^]^ It has also been reported that the capacitance varied with the change of the pore size.^[^ ^72^ ^]^ These findings suggest that there is no simple correlation between the specific capacitance and the specific surface area. Such fundamental principles of double‐layer behavior and charge storage mechanisms in porous electrodes are in urgent need of further work.^[^ ^65^ ^]^

In the pseudocapacitance case, surface Faradaic redox reactions and intercalation–deintercalation of cations in active materials both contribute to the capacitance (Figure 4). In Conway's explanation of the surface redox charge storage mechanism, reversible redox reactions are believed to occur at or near the surface of appropriate electrode materials, whereas the electrochemical characteristics are similar to those of EDLCs, but with considerably greater charge storage because of the redox reactions.^[^ ^73^ ^]^ The characteristic feature of the pseudocapacitive mechanism is the variation of charge with potential along with the absence of solid‐state diffusion limitation and phase change of the material.^[^ ^65^ ^]^

MXenes are among the most intensively reported 2D pseudocapacitive materials. Taking Ti_3_C_2_T_*x*_ as an example, the valence change of surface Ti atoms (Ti^2+^/Ti^3+^) is balanced by the reversible insertion–deinsertion of protons, which is different from the bulk Faradaic storage.^[^ ^74^ ^]^ This charge storage behavior displays a rectangular‐shaped cyclic voltammetry (CV) curve, which is similar to that of EDLCs.^[^ ^75^ ^]^ In addition, the electrolyte was reported to have a tremendous impact on the ion intercalation–deintercalation process, which is directly related to the charge storage.^[^ ^75^ ^]^ A high capacity of 130 mAh g^−1^ was obtained when the electrolyte of lithium bis(trifluoromethanesulfonyl)imide (LiTFSI) in propylene carbonate (PC) was used due to the complete desolvation of Li ions inserted between MXene layers. Nevertheless, replacing the PC solvent by acetonitrile and dimethyl sulfoxide caused a decrease in capacitance, because Li ions drove solvent molecules into the interlayer at the time of intercalation.^[^ ^75^ ^]^ The lesson to be learned from this is that improving supercapacitor performance by optimizing charge storage mechanisms always faces a complex interplay of various processes.

### 3.2 Fundamental Electrochemistry in Lithium Batteries

In a lithium battery cell, phase transformation, interfacial reaction, charge storage, and performance degradation occur during the operation. This complex interplay must be analyzed to understand and design lithium batteries.

#### 3.2.1 Transformations of Materials during Operation

Lithium battery electrodes usually consist of a mixture of electroactive components, conductive materials, and polymer binder. During the charge/discharge of a battery, the electrode materials interact with the electrolyte resulting in marked transformations that manifest themselves in several ways, such as the change of structural phase, morphology, oxidation state, etc. Depending on the electrode materials, different transformation processes, such as alloying, conversion reaction, and intercalation, may occur.^[^ ^76^, ^77^ ^]^ Metals or semimetals can form alloys with lithium electrochemically, giving rise to a high capacity. However, this alloying reaction is usually accompanied by huge volume changes, resulting in progressive decohesion, particle reorganization, and subsequent capacity loss.^[^ ^77^ ^]^ Another mode of charge storage is through the reversible conversion mechanism, when under electrical bias the active materials react with the electrolytes to form completely new products with different structures and chemistry. Transition metal oxides and TMDs are representative materials that operate through this mechanism.^[^ ^78^ ^]^ It is worth mentioning that there are reports about 2D materials such as InSe nanoflakes that exhibit both conversion and alloying mechanisms of charge storage.^[^ ^79^ ^]^ As expected, layered InSe is prone to extreme volumetric changes. Nanoflakes exfoliated from bulk InSe when mixed with carbon nanotube could effectively tackle this. Quite surprisingly, over several hundred cycles of alloying/dealloying In gave rise to the formation of tiny nanoclusters, allowing it to accommodate 4 Li ions instead of 1, achieving capacity higher than theoretically predicted values. From the operando characterizations of this system, it was suggested that InSe during the first cycle was irreversibly converted into metallic In hosted on Li_2_Se nanoflakes, whereas in the subsequent cycles, an In alloying mechanism dominated. This example suggests that when multiple mechanisms are involved, in situ and operando techniques become particularly important to distinguish their individual contribution. Finally, we have the intercalation mechanism in lithium‐ion batteries, the history of which goes back to the 1970s.^[^ ^80^, ^81^ ^]^ The first intercalation‐type batteries consisted of carbon as the anode and LiCoO_2_ as the cathode, and their redox reactions with lithium were based on intercalation. For most electrodes made from 2D materials with expanded interlayers, the intercalation of Li ions leads to improved performance. For example, Liu and co‐workers piled up MoS_2_ layers along the *c*‐axis via preliminary exfoliation and subsequent restacking. Then, the enlarged interlayer distance of obtained MoS_2_ favored the intercalation process, giving rise to high capacity and long cycling life.^[^ ^82^ ^]^

#### 3.2.2 Interfacial Electrochemistry of Electrode and Electrolyte

**Figure 5:**
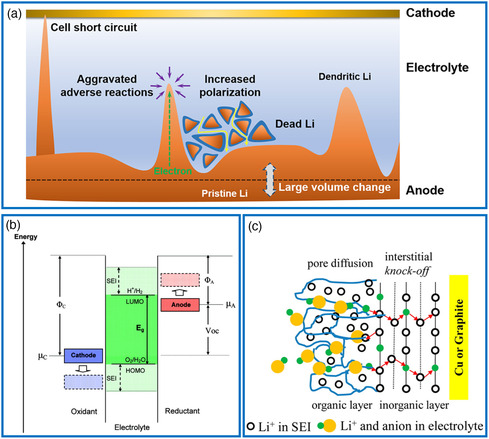
Schematic illustration of some important processes in lithium batteries. a) Formation of Li dendrites in rechargeable batteries. Reproduced with permission.^[^ ^83^ ^]^ Copyright 2017, American Chemical Society. b) SEI formation condition in a liquid electrolyte. Reproduced with permission.^[^ ^66^ ^]^ Copyright 2009, American Chemical Society. c) Diagram of SEI composed of porous organic layer and dense inorganic layer. Reproduced with permission.^[^ ^84^ ^]^ Copyright 2012, American Chemical Society.

The interfacial properties of electrode–electrolyte are pivotal for the device performance and safety of lithium batteries. Typical interfacial phenomena are lithium dendrite growth, electrolyte degradation, gas evolution, and solid electrolyte interphase (SEI) formation. The occurrence of Li dendrites at the interface caused by the high chemical reactivity of Li leads to a short circuit of the working cell, increased polarization, and large volume changes. These phenomena are responsible for critical safety issues, reducing cycle life, and obtaining low coulombic efficiency (**Figure** **5**a). The SEI as proposed by Goodenough et al. has a close relationship with the highest occupied molecular orbital (HOMO) and the lowest unoccupied molecular orbital (LUMO) of electrolytes (Figure 5b). If an anode with electrochemical potential (denoted as *μ*_A_) lies above the LUMO energy, i.e., *μ*_A_ > *E*_LUMO_, then electrons will transfer from the anode to the unoccupied orbital of the electrolyte. This reduces the electrolyte until the SEI layer forms as a barrier to inhibit further electron transfer.^[^ ^66^ ^]^ Similarly, in a cathode with electrochemical potential (denoted as *μ*_c_) below the HOMO energy, the oxidation of the electrolyte will continue until the passivated SEI formation protects it from further exfoliation or corrosion. Thus, a perfect SEI is required having the proper thickness, high ionic conductivity, strong mechanical performance, and pronounced chemical stability to furnish good performance and high lifetime of batteries.^[^ ^83^ ^]^ It has been reported, however, that the SEI layer is not homogeneous throughout its thickness, but rather forms a dual‐layer structure of outer organic layer and inner inorganic sheets (Figure 5c).^[^ ^84^ ^]^ The SEI may contain a complex blend of inorganic compounds such as Li_2_O, LiF, and LiOH near the interface as well as organic compounds with higher oxidation states such as ROCO_2_Li, ROLi, and RCOO_2_Li (R denotes the organic group) farther away.^[^ ^83^, ^85^ ^]^ However, the precise composition and morphology of SEI are complex and quite diverse for different batteries, so that the exploration of SEI has attracted a lot of attention. In addition, various strategies for stabilizing the interface have been proposed, including modification of the electrolyte compositions and design of nanostructured or coated electrodes.^[^ ^86^, ^87^, ^88^, ^89^, ^90^, ^91^ ^]^

## 4 In Situ and Operando Techniques Applied to 2D Model Energy Storage Devices

The enormous value of in situ and operando techniques has already been emphasized earlier. Recently, tremendous progress has been made toward the development of advanced in situ characterization techniques, each of which has unique capabilities to study specific properties or progress.^[^ ^15^ ^]^

Among a plethora of X‐ray‐based characterization tools, X‐ray diffraction (XRD), X‐ray absorption spectroscopy (XAS), and X‐ray photoelectron spectroscopy (XPS) are the most frequently used ones. A main concern with the in situ X‐ray‐based techniques is the reflection and absorption of X‐rays by nontargeted materials, leading to the weakness of the incident X‐ray intensity, which further affects the acquired data quality.^[^ ^92^ ^]^ On the other hand, high beam energy may promote secondary reactions due to the occurrence of beam‐induced reactions. For in situ XPS characterization, the production of insulating materials during the observation process will also cause charging problems for the calibration and data acquisition.^[^ ^92^ ^]^ IR spectroscopy (IR absorption and IR reflectivity) and Raman scattering are two of the most versatile optical characterization tools that provide real‐time information about chemical changes during operation. Care should be taken to the selection of radiation sources to balance a good signal‐to‐noise ratio without local heating or sample damage and to avoid the fluorescence effect in Raman.^[^ ^93^ ^]^ Nuclear magnetic resonance (NMR) based on the interaction of magnetic moments of nuclei of various atoms with magnetic fields is a powerful tool for the investigation of interfacial processes.^[^ ^94^ ^]^ The factors, such as bulk magnetic susceptibility, magnetic field orientation, sample orientation to the magnetic field, skin effect or skin depth, and the direction of the materials, pose difficulties in getting in situ NMR spectra.^[^ ^95^ ^]^ Microscopy follows the morphological evolution at various length scales, among which optical microscopy (OM) can provide a bird's‐eye view of morphology evolution, whereas atomic force microscopy (AFM) allows a closer look. The major challenges for in situ AFM setup design are the approach of the cantilever to target materials and the inertness to electrochemistry. By utilizing electron beams, scanning electron microscopy (SEM) and transmission electron microscopy (TEM) furnish high magnifications to observe the minute details of the morphological changes. Special attention should be paid for the tested sample with an electron‐based analysis, which should not react in the electron beam and should remain stable in high vacuum conditions to avoid side reactions or even artifacts. Sometimes, they are coupled with spectroscopic tools to perform the elemental analysis of nanosized entities.

These characterization techniques have been discussed in detail in many previous reviews and need not be described in detail here.^[^ ^15^, ^17^, ^92^ ^]^ For reliable in situ and operando measurements, model devices with a special cell design are needed. An ideal electrochemical model device for in situ and operando characterization should be easily observed and represents a “real” energy storage device. Therefore, significant efforts have been made to develop unique cell configurations and model structures using 2D materials for experimental techniques, enabling in situ and operando investigation of electrochemical reaction mechanisms.^[^ ^96^ ^]^ The essence here is the use of in situ and operando analysis of energy storage in 2D materials to provide suggestions for future efforts.

### 4.1 Typical Examples of Materials and Processes Studied in Supercapacitors

It is generally accepted that nanostructures of materials have a profound effect on capacitance by sometimes demonstrating unexpected storage mechanisms. Historically, carbon has been the most widely used material for supercapacitors, so most of the early in situ and operando investigations attempted to understand its peculiar behavior under different circumstances. IR spectroscopy, providing information on molecular composition and structure,^[^ ^97^ ^]^ and NMR, providing molecular level, quantitative, and element specific information,^[^ ^94^ ^]^ have been used to reveal the structure of the electrical double layer in supercapacitors, pore size influence in performance, and the possible mechanisms of charge storage.^[^ ^16^, ^98^, ^99^, ^100^ ^]^

**Figure 6:**
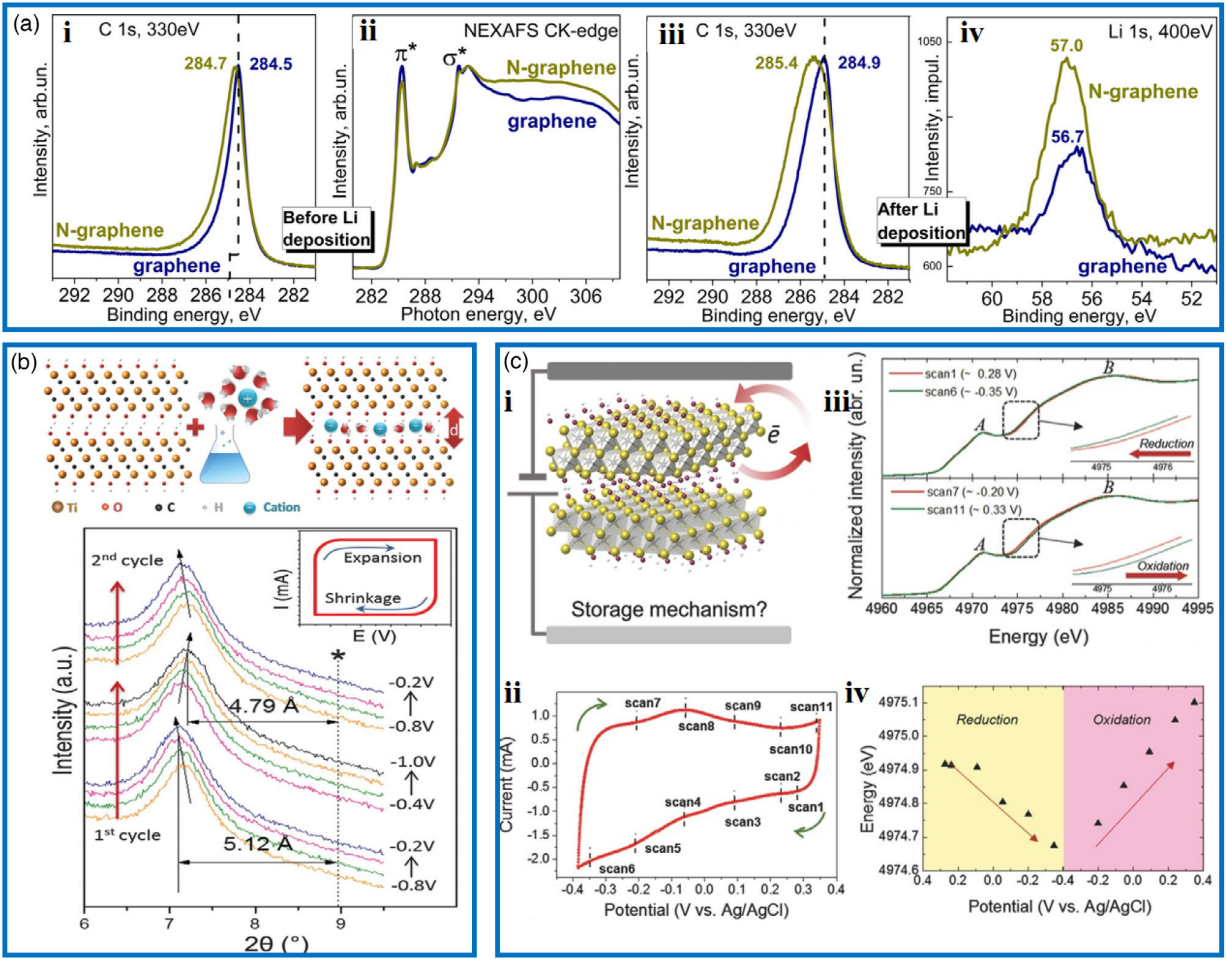
Typical examples of supercapacitors studied by in situ and operando techniques. a‐i) C 1s spectra and ii) C K‐edge spectra of annealed graphene and N‐doped graphene films on SiO_2_/Si substrates. iii) XPS C 1s and iv) Li 1s spectra of graphene and N‐doped graphene films after the deposition of lithium. a) Reproduced with permission.^[^ ^105^ ^]^ Copyright 2017, American Chemical Society. b) Schematic description of the intercalation of cations between Ti_3_C_2_T_*x*_ layers. Electrochemical in situ XRD study of Ti_3_C_2_T_*x*_ layers in 1 m potassium hydroxide (KOH) solution. b) Reproduced with permission.^[^ ^108^ ^]^ Copyright 2013, American Association for the Advancement of Science. c) Electrochemical in situ XAS data. i) Schematic description of the Ti_3_C_2_(OH)_2_ structure and possible charge transfer processes. ii) CV curves collected during in situ XAS detection in 1 m H_2_SO_4_ electrolyte. iii) Ti K‐edge XANES spectra. iv) Variation of Ti edge energy as a function of potential. c) Reproduced with permission.^[^ ^110^ ^]^ Copyright 2015, Wiley‐VCH.

Doping of traditional materials can greatly improve their performance. N‐doped carbon materials generally show high performance as electrode compared with their N‐free carbon counterparts.^[^ ^101^, ^102^ ^]^ The XPS method, which is one of the most widely used surface characterization methods across many disciplines,^[^ ^103^ ^]^ can offer information about the element composition and chemical state and has been widely utilized to follow elementary electrochemical reaction steps at electrode surfaces.^[^ ^104^ ^]^ Model experiments studied with in situ XPS were performed by Bulusheva et al. to explore the difference in the interaction of lithium with graphene and N‐doped graphene, in which both kinds of graphene were prepared through CVD growth on Cu foil and transferred onto SiO_2_/Si substrates.^[^ ^105^ ^]^ The deposition of Li onto both films was carried out in a vacuum chamber of the X‐ray spectrometer. The comparative analyses of the obtained C 1s and Li 1s spectra before and after Li deposition revealed that the N‐doped graphene with a larger shift of C 1s spectrum interacted more strongly with Li than the pure graphene, which then enabled stronger adsorption of Li (**Figure** **6**a).

MXenes have shown promise as electrode materials for supercapacitors with high capacitance. An intercalation‐induced high capacitance has been revealed by combining in situ XRD techniques with electrochemical characterizations.^[^ ^74^ ^]^ XRD is based on the scattering of X‐rays, the interference of which produces diffraction patterns from crystalline or partially crystalline materials.^[^ ^106^, ^107^ ^]^ Thus, the in situ and operando XRD technique is suitable to monitor crystal structure changes of the electrodes during the charging and discharging process. Gogotsi and co‐workers reported the spontaneous intercalation of monovalent cations of Li^+^, Na^+^, K^+^, NH_4_^+^, and multivalent cations of Mg^2+^, Al^3+^ between the 2D Ti_3_C_2_T_*x*_ layers (schematic description of Figure 6b), leading to an increase in the interlayer spacing.^[^ ^108^ ^]^ In this work, in situ XRD results (Figure 6b) indicated that the *c* values fluctuated within 0.33 Å, as the potential changed when the Ti_3_C_2_T_*x*_ electrode was tested in the 1 m KOH solution electrolyte. It is also observed that a slight shrinkage was presented in the interlayer distance upon increasing the voltage. This phenomenon was observed even with other aqueous electrolytes such as NaOAc, MgSO_4_, 1‐ethyl‐3‐methylimidazolium bis(trifluoromethyl‐sulfonyl)imide ionic liquid, and organic electrolytes.^[^ ^109^ ^]^ This shrinkage may be of electrostatic origin (or steric in the presence of large intercalating ions). X‐ray absorption near‐edge structure (XANES) interrogates an electronic structure and symmetry of the metal site, and thus, the in situ XANES was also applied to shed light on the mechanism of capacitance in Ti_3_C_2_T_*x*_ MXene.^[^ ^110^ ^]^ During the charge and discharge process, Ti K‐edge XAS spectra were collected, as shown in Figure 6c. It can be seen from the CV curve and the XANES spectra that the Ti edge has shifted to low energy with the sweep from high (0.275 V) to low (‐0.35 V) potential, indicating a decrease in the average oxidation state of Ti atoms. Accordingly, the XANES spectrum shifted back to the higher energy, representing a higher oxidation state, during the reverse scan. Through careful analysis, it is concluded that the electrochemical behavior of Ti_3_C_2_T_*x*_ in 1 m H_2_SO_4_ electrolyte was predominantly pseudocapacitive.

### 4.2 Examples of Materials and Processes Studied in Lithium Batteries

**Figure 7:**
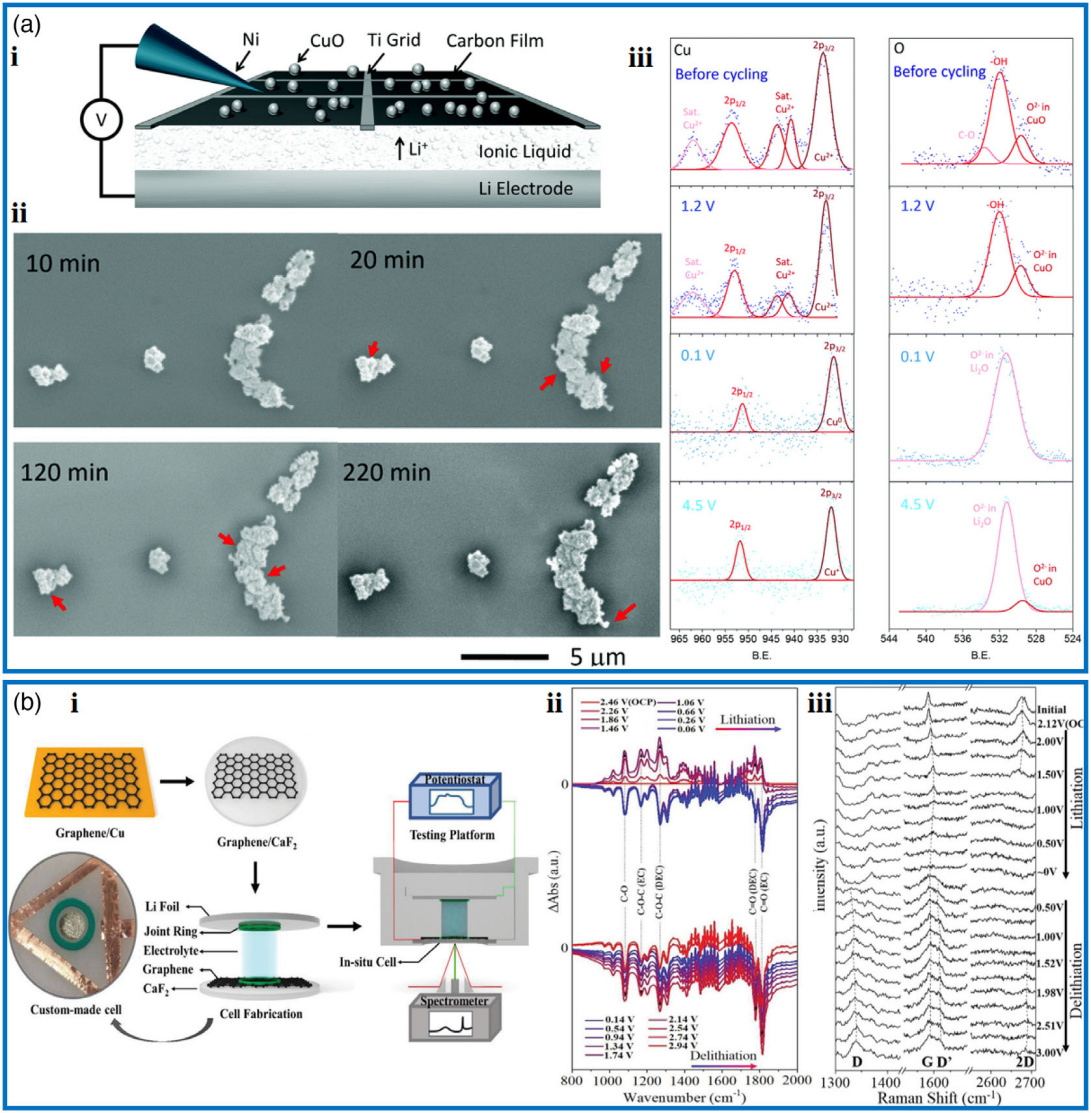
Typical examples of batteries with open cells studied by spectroscopy techniques. a‐i) Schematic representation of the particular cell for in situ characterization. ii) Sequences of in situ SEM images of CuO nanoparticle agglomerates under a galvanic charging to 0.1 V. iii) In situ XPS spectra of the Cu 2p and O 1s at different stages. a) Reproduced with permission.^[^ ^112^ ^]^ Copyright 2016, The Royal Society of Chemistry. b‐i) Schematic illustration of the transformation of SLG and in operando cell for Raman and FTIR observations. ii) In situ FTIR spectra and iii) in situ Raman spectra of SLG during the first electrochemical cycle. b) Reproduced with permission.^[^ ^117^ ^]^ Copyright 2019, Wiley‐VCH.

In situ battery characterization follows two general approaches of open cells and closed cells. Closed cells are the most ideal representation of commercial batteries, but only provide limited information for the mean free path of the source and signal exceed that of the electrochemical cell size. Open cells offer large room for manipulation, measurement, and characterization of the cycled material.^[^ ^111^ ^]^ Dillon et al. designed an open cell composed of low saturation vapor pressure ionic liquid electrolyte, Li permeable 2D amorphous carbon membrane supported by a metallic grid, and Li electrode (**Figure** **7**a). With the in situ XPS and SEM characterization of the evolution of structure, composition, and bonding during cycling, the advantage of the open electrochemical cell approach to cross platform in situ characterization could be convincingly demonstrated.^[^ ^112^ ^]^

The phase transformation of the electrode can be monitored by in situ and operando Raman and IR spectroscopy, both of which are complementary in nature and together provide comprehensive chemical analysis of a given system.^[^ ^113^, ^114^ ^]^ For example, the phase transition process from LiC_72_ (dilute stage 1) to LiC_6_ (stage 1) via three charging plateaus has been verified by in situ Raman spectra.^[^ ^115^ ^]^ The splitting of G peak and shift of 2D peak in the Raman spectra of graphite have been reported to reflect the stage of Li intercalation into graphite.^[^ ^116^ ^]^ In situ Fourier transform infrared spectroscopy (FTIR) and Raman spectroscopy were combined by Zhu and co‐workers for single‐layer graphene (SLG) grown from CVD and transferred to CaF_2_ crystal (Figure 7b–i).^[^ ^117^ ^]^ Through small changes of C—O, C—O—C, and C=O signals in FTIR reflectivity spectra during the electrochemical cycles, it was found that the SEI formed in the first cycle and stabilized in the second and third cycles (Figure 7b‐ii). The shift of D, G, and 2D peaks in Raman revealed the structural evolution of SLG (Figure 7b‐iii). Combining ex situ OM, SEM, TEM, and the theoretical calculation, it was shown that the adsorption/desorption of Li ions on SLG readily induced defects for further adsorption of Li. The avalanche deposition resulted in much more defects in SLG covered by SEI containing Li species. Although the SLG hardly qualifies as a “practical” electrode, it is illustrative to subject it to in situ characterizations to differentiate and compare the processes that arise on a single layer with those occurring in stacked multiple layers.

**Figure 8:**
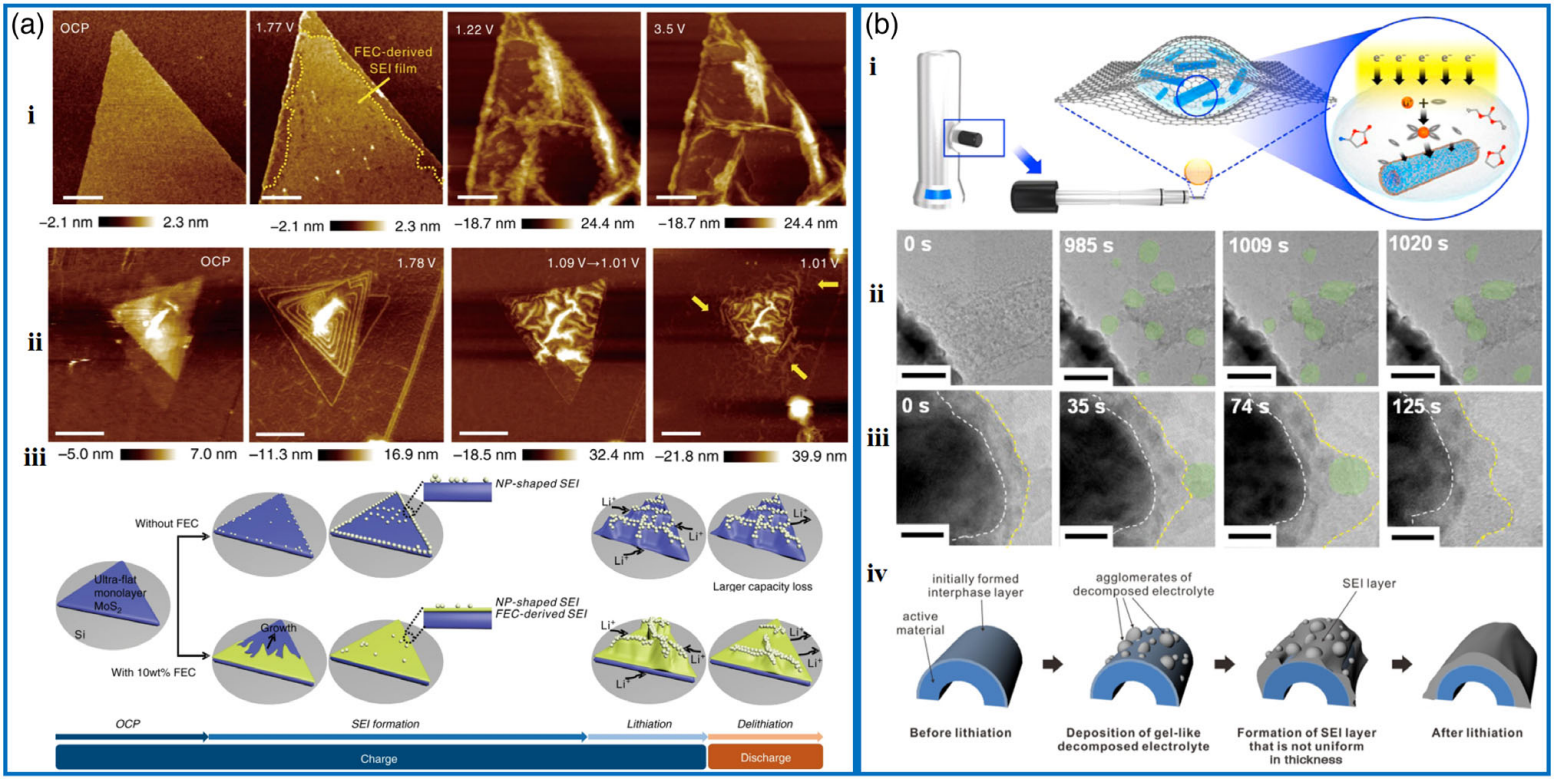
SEI formation studied by in situ AFM and TEM. a) In situ AFM images of i) FEC‐derived SEI formation and of ii) defect‐induced structural evolution on a MoS_2_ electrode at marked potentials. iii) Schematic representation of the structural evolution and reaction mechanism of the MoS_2_ electrode. a) Reproduced under the terms of the CC‐BY 4.0 license.^[^ ^123^ ^]^ Copyright 2019, The Authors, published by Springer Nature. b‐i) Schematic description of the in situ TEM observation of special GLC. ii,iii) HRTEM images showing the agglomeration of electrolytes and formation of SEI layer, respectively. The scale bars in (ii) and (iii) are 100 and 10 nm , respectively. iv) Schematic illustration of the suggested formation of SEI layer. b) Reproduced with permission.^[^ ^125^ ^]^ Copyright 2016, Elsevier Ltd.

The characterization of SEI layer formation during (de)lithiation helps to understand the stability of the interface, which has been probed by in situ techniques with model 2D materials.^[^ ^92^, ^118^, ^119^, ^120^, ^121^, ^122^ ^]^ Wan et al. studied the SEI evolution and the lithiation/delithiation process on the monolayer MoS_2_ electrode by in situ electrochemical AFM.^[^ ^123^ ^]^ The results revealed that the fluoroethylene carbonate (FEC)‐containing electrolyte and local defects affected the morphology change and electrochemical performance. The SEI structure evolution observed by in situ AFM disclosed that the FEC‐containing electrolyte was conducive and formed a dense and uniform SEI layer in the interface, thus protecting the electrode from side reactions and volume expansion (**Figure** **8**a–i). The presence of localized defects such as the step edges facilitates Li^+^ accommodation, thereby enhancing the reaction kinetics (Figure 8a‐ii). Figure 8a‐iii is a schematic diagram showing the structural evolution due to the synergistic effect between the electrolyte and the anode material. Besides, the appearance of wrinkles upon lithiation reveals the intrinsic flexibility of MoS_2_, whereas the retention of wrinkles upon delithiation clearly explains the capacity degradation of the MoS_2_‐based lithium batteries.

TEM, where electrons transmitted through the sample are focused by electromagnetic lenses and collected by a detector to form an image,^[^ ^124^ ^]^ has also been utilized to explore the evolution of SEI. For instance, Kim and co‐workers designed the special graphene liquid cell (GLC) composed of two graphene sheets and encapsulated electrolyte, which is easy to be fabricated and offers high resolution due to high electron transport rate (Figure 8b–i).^[^ ^125^ ^]^ SnO_2_ as the active material is immersed in the electrolyte. The in situ high resolution TEM (HRTEM) images in Figure 8b‐ii,iii show the diffusion of Li into SnO_2_ during the decomposition of electrolytes; meanwhile, the uneven layer forms initially and undergoes stabilization to develop a more uniform SEI layer. With the confirmation from ex situ HRTEM results, it is proposed that the SEI formation process contained three main processes of initial formation, agglomeration, and stabilization illustrated by the scheme in Figure 8b‐iv.

Insight into the evolution and arrangement of electrolyte during the cycling is the key step to understand the energy storage mechanism. Smet and co‐workers studied the reversible intercalation of lithium into bilayer graphene by in situ low‐voltage TEM with an on‐chip architecture to prevent disturbance of graphene due to electrolyte.^[^ ^126^ ^]^ Based on the obtained data, the authors proposed the new form of a close‐packed Li phase between the two graphene layers, which was also supported by the electron energy loss spectroscopy and density functional theory (DFT) simulations. During lithiation, the close‐packed Li phase rapidly diffused laterally between the graphene layers at a rate of ≈1 Å s^−1^, whereas the close‐packed Li phase disassembled and disappeared gradually during delithiation. Such in situ characterization of transport properties along with the structural evolution can provide a comprehensive picture of different mechanisms of 2D materials in general and also highlight the specifics of individual materials.

Examination of the structural changes and interaction with cathode materials of sulfur species has played a crucial role in understanding the reaction processes in lithium–sulfur batteries.^[^ ^127^, ^128^, ^129^, ^130^ ^]^ The morphological changes and phase variations of the sample can be observed with the in situ OM, which is noninvasive and simply relies upon illumination of the sample. For example, Cui and co‐workers conducted in situ studies of the electrochemical deposition of sulfur on 2D MoS_2_ layers with the design of the optical cell.^[^ ^131^ ^]^ The cell was made up of MoS_2_ as the working electrode, lithium as the counter electrode, and polysulfides in organic solvent as the electrolyte. The snapshots from the in situ OM video demonstrate the sulfur generation on MoS_2_ during charging to 3.0 V, which reveal the formation of solid sulfur at the edges, whereas liquid sulfur droplets grow on the basal plane at first. The solid sulfur quickly expands across the basal plane and covers the whole surface of MoS_2_ with further growth. A similar sulfur growth was observed on other 2D materials, including WS_2_, MoSe_2_, WSe_2_, and graphite. In situ Raman and in situ XANES were also conducted to confirm that the amorphous liquid droplets were composed of sulfur, and that the S_8_ molecules in solid sulfur exhibited long‐range order. By combining this experimental characterization with DFT calculations, the authors explained the distinct growth features as being influenced by a variety of factors, including binding energy, wetting, electric field distribution, and critical nucleus size. In addition, it is argued that the liquid sulfur furnished a higher capacity than the solid sulfur under the same charge condition.

## 5 Conclusion and Outlook

During the past decade, great progress has been made in developing and applying 2D materials in supercapacitors and lithium batteries owing to their unique physicochemical properties. Nevertheless, many processes such as the electrochemical reaction, degradation, and thermal decomposition under realistic operation conditions remain undisclosed. A deeper understanding of these important electrochemical phenomena during charging and discharging is essential for further improvement of energy storage devices. Many advanced in situ and operando characterization techniques provide unprecedented insights into the structural, morphological, and chemical characteristics of electrodes, electrolyte, electrolyte–electrode interface, and their correlations with the electrochemical performance. The 2D materials have opened a new chapter of energy storage, because they allow straightforward preparation and operation. Furthermore, they provide access to ideal model structures suitable for in situ characterization and mechanistic analyses. However, some challenges and emerging opportunities should be considered.

1) The 2D materials have been proved as extremely promising electrode materials for electrochemical energy storage, and there is no doubt that further exploration and application of novel 2D materials will continue to attract attention of researchers. Despite a large number of 2D material families synthesized in fundamental research, their scalable fabrication is still elusive. 2) The application of practically relevant in situ and operando techniques for supercapacitors and lithium battery systems is still in its infancy. Some systems have not been studied by in situ techniques at all, especially those with liquid electrolytes. As concerns EDLCs, the migration, adsorption, and exchange of ions are still difficult to monitor. Therefore, the combination of experimental approaches with dynamics simulations is supposed to provide fresh insights. 3) One should not forget that there is still a gap of knowledge between model devices for in situ studies and “real” devices. On the one hand, more creativity in designing technologies for in situ cells is needed. On the other hand, further development of new analytical approaches is essential, which should be noninvasive and allow realistic device fabrication and operation.^[^ ^132^ ^]^ Clearly, advanced light sources such as synchrotron radiation and lasers offer special advantages for future in situ characterization. 4) A specific technique can only provide limited information; thus, spectroscopy offers more chemical information, whereas microscopy monitors the morphology. It is necessary to integrate multiple characterization techniques and to target a unified picture of the underlying mechanisms, which are often difficult to implement because of the different experimental procedure and cell requirements of different techniques. 5) Experiments investigated by in situ and operando techniques often produce amounts of data, from which the dynamic phenomena of interest are obvious sometimes, but more commonly, extracting important aspects of dynamic process is a difficult task. Therefore, improved data analytic could be a boon to the development of in situ and operando experiments, where the intelligent analysis can reduce the complexity of the full data set and provide information regarding regions or events of interest, and even can be performed in real time to guide the scientist. 6) Finally, these in situ and operando techniques hold enormous promise for other electrochemical systems, such as sodium, potassium, aluminum, and zinc ion batteries, which demonstrate the general importance of these developments.

## Conflict of Interest

The authors declare no conflict of interest.
